# Odors modulate color appearance

**DOI:** 10.3389/fpsyg.2023.1175703

**Published:** 2023-10-06

**Authors:** Ryan J. Ward, Maliha Ashraf, Sophie Wuerger, Alan Marshall

**Affiliations:** ^1^Department of Computer Science and Mathematics, Liverpool John Moores University, Liverpool, United Kingdom; ^2^Department of Electrical Engineering and Electronics, University of Liverpool, Liverpool, United Kingdom; ^3^Department of Psychology, University of Liverpool, Liverpool, United Kingdom; ^4^Department of Computer Science and Technology, Cambridge University, Cambridge, United Kingdom

**Keywords:** crossmodal correspondences, crossmodal associations, odors, olfaction, colors, neutral gray, color perception, olfactory perception

## Abstract

Our brain constantly combines multisensory information from our surrounding environment. Odors for instance are often perceived with visual cues; these sensations interact to form our own subjective experience. This integration process can have a profound impact on the resulting experience and can alter our subjective reality. Crossmodal correspondences are the consistent associations between stimulus features in different sensory modalities. These correspondences are presumed to be bidirectional in nature and have been shown to influence our perception in a variety of different sensory modalities. Vision is dominant in our multisensory perception and can influence how we perceive information in our other senses, including olfaction. We explored the effect that different odors have on human color perception by presenting olfactory stimuli while asking observers to adjust a color patch to be devoid of hue (neutral gray task). We found a shift in the perceived neutral gray point to be biased toward warmer colors. Four out of five of our odors also trend toward their expected crossmodal correspondences. For instance, when asking observers to perform the neutral gray task while presenting the smell of cherry, the perceptually achromatic stimulus was biased toward a red-brown. Using an achromatic adjustment task, we were able to demonstrate a small but systematic effect of the presence of odors on human color perception.

## 1. Introduction

In our everyday life, we are simultaneously bombarded with information from different sensory modalities. Our brain combines this information to better understand our surrounding environment (Calvert et al., 2005); this integration process has been shown to influence our perception in different senses, for example (Morrot et al., 2001). Crossmodal correspondences is the tendency for a sensory attribute to be associated with a stimulus feature in a different sensory modality (Spence, 2011; Spence and Parise, 2012). For instance, people have consistent correspondences between odors and a variety of different sensory modalities, including but not limited to the angularity of shapes, the smoothness of texture, pitch, colors, and musical dimensions (Ward R. J. et al., 2021). These correspondences still occur outside of olfaction, including, but not limited to, sound-taste (Knöferle and Spence, 2012), temperature-color (Motoki et al., 2019), and pitch-vertical position (Bernstein and Edelstein, 1971). The nature and origin of these correspondences have diverse characterization in the literature with hedonics (Crisinel et al., 2013; Hanson-Vaux et al., 2013), semantics (Demattè et al., 2006; Jacquot et al., 2016), and natural co-occurrence (Spence, 2011; Spence and Deroy, 2013) being frequently deducted.

Colors have a profound effect on the perception of odors (Zellner and Kautz, 1990; Zellner and Whitten, 1999). For instance, observers may perceive an orange-flavored drink if it is colored orange when in fact, it is cherry-flavored (DuBose et al., 1980). A later study by Morrot et al. (2001) has shown that this effect can bias the judgment of expert wine tasters. That is when a glass of white wine is artificially colored red with an odorless dye; a panel described it as a red wine. Crossmodal correspondences are presumed to be bidirectional in nature (Evans and Treisman, 2010), meaning if odor-color correspondences are strong enough, one could expect the presence of odors to slightly influence our color perception. A study by Hansen et al. (2006) found that when asking observers to adjust the color of fruit objects to appear achromatic, participants overcorrected the color stimuli to the opponent color of the presented visual aid. That is, when asking their observers to adjust the color of a banana to a neutral gray, the observers adjusted the banana to a “blueish hue,” which is the opponent color of yellow. Hansen et al. (2006) state that this is due to a visual memory modulation effect, meaning that knowledge of the color that corresponds with the identity of visual cue (e.g., banana being associated with the color yellow) would modulate the color appearance.

The existence of stable and strong crossmodal correspondences between odors and colors may reflect interactions taking place at a perceptual level (Demattè et al., 2009). For instance, olfactory crossmodal correspondences have recently been shown to be predictable based on their physical and chemical characteristics (Ward R. et al., 2021; Ward et al., 2022). Kemp and Gilbert (1997) asked participants to select color chips that best matched the presented odor. They found that the weaker the perceived intensity, the lighter the selected colors. Kemp and Gilbert (1997) state that this finding could be attributed to the interaction taking place at a perceptual level rather than a decisional (see also Gilbert et al., 1996). However, it is important to note the importance of semantic information and language in the nature and origin of odor-color correspondences (see de Valk et al., 2017; Kaeppler, 2018; Ward R. J. et al., 2021). In terms of olfactory discrimination and identification, color (Zellner et al., 1991; Narumi et al., 2010) and other visual information play an important role (Demattè et al., 2009).

In our prior work (Ward R. J. et al., 2021), we uncovered the crossmodal correspondences between odors and the angularity of shapes, smoothness of texture, pitch, colors, perceived pleasantness, and emotional/musical dimensions. Additionally, we probed the nature and origin of these correspondences. We found that the knowledge of an odor’s identity (semantics) plays a role when judging the emotional and musical dimensions as well as color correspondences. Hedonics was also found to be the main mediator for the angularity of shapes, smoothness of texture, pleasantness, and pitch correspondences. Of particular interest for this manuscript is color correspondences where we found robust crossmodal correspondences between odors and colors, see Ward R. J. et al., (2021; Figure 3B). We used a subset of olfactory stimuli used in Ward R. J. et al. (2021) that induced the most consistent odor-color correspondences (caramel, cherry, coffee, lemon, and peppermint); in addition to these odors, we added a control (plain unscented water). Here we hypothesize that the presence of odors would influence the perception of color. Based on the findings of Hansen et al. (2006), we anticipated that when asking the observers to create a color patch that is achromatic (a neutral gray), the findings would reveal a shift to a color that is opposite to the one associated with the particular odor. That is, if the presented odor is primarily associated with the color yellow, we anticipate that the perceived neutral gray point would be shifted more toward blue.

## 2. Methodology and materials

### 2.1. Participants

Twenty-four people participated in the experiment (eleven males and thirteen females). The mean age of the participants was 29.12. The youngest participant was 20 years of age, and the oldest was 57 years of age. No participants reported any impairment affecting their sense of smell (i.e., cold, flu, COVID) or color vision (i.e., color blindness). Participants were briefed before the experiment started on potential allergens and the task. Participants were instructed not to wear any scented deodorants, or perfumes on the day of the experiment. Color vision was not tested before the observer’s participation and was assumed based on the observer’s report. The experiment was approved by the University of Liverpool and conducted in accordance with the declaration of Helsinki’s standard for medical research involving human subjects. Observers were recruited via mass e-mail. The sample size for this study was considered from a variety of resources. For the circular statistics, a sample size of at least 20 is required (NCSS, 2010). To estimate the sample size needed for the rest of the statistics, *a priori* statistical power analysis was performed in G*Power. For a one-tailed *t*-test with a power of 0.9, α = 0.05 and a computed effect size of 0.66, a sample size of at least 22 is required.

### 2.2. Experimental conditions

**Figure 1:**
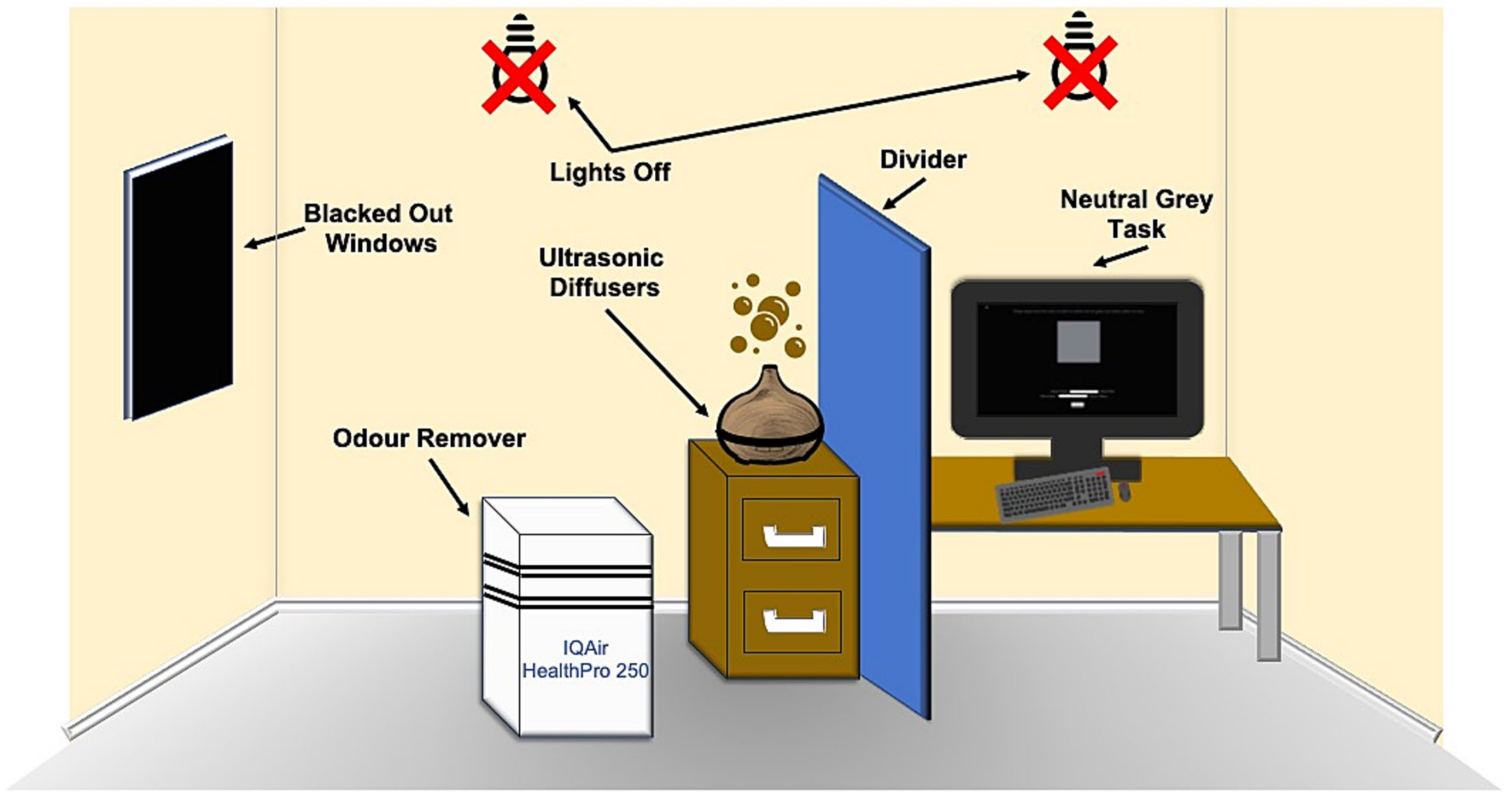
Diagram of the experimental setup.

The windows in the room were blacked out using blackout window film and the lights were turned off during the experiment, to ensure consistent illumination. The observers were given 5 min to adjust to the dim light before starting the experiment. The task was performed in full-screen mode, to stop the observers from using a color located elsewhere on the screen to aid in their decision. This was to stop observers from using colors elsewhere on the screen (i.e., white text) in an endeavor to make a neutral gray. The task was conducted in four parts. The first part consisted of odor removal using an IQAir HealthPro 250 Air Purifier that lasted 4 min and was done even before the first odor was presented to the observer. In the second part, ambient odor was introduced into the experiment room using ultrasonic diffusers for 5 min. In the third part, the observer was asked to adjust a color patch, so that it was devoid of any hue (neutral gray task). In the fourth part, observers were asked to identify the odor that was being presented (classification task). This process was repeated until the observer had completed all four parts for each one of the four odors and the control. The timings for these parts were determined by a small test of five participants. This test involved running through a reduced version of the experiment, where only the odors were presented to the participants and involved noting down the timings for when either they could smell the odor that was being released or when they felt they could no longer smell the last presented odor. The largest timing across odors was used as the measurement for how long to present each odor and the largest timing for the smell evacuation process was used. Additionally, a minute was added to these timings. These timings are the timings used in the main experiment. The odors were presented in a random order to the participants in accordance with a number displayed on the screen. This number was an identifier, so the experimenter knew which odor to present and when. The color patch was adjusted five times for each condition. The diffusers were cleaned, and new smells were added each time the experiment was conducted. Diffusers were used for this experiment as a COVID safety measure. The experiment was programmed in MATLAB R2021b. See Figure 1 for a diagram of the experimental setup.

### 2.3. Neutral gray task

**Figure 2:**
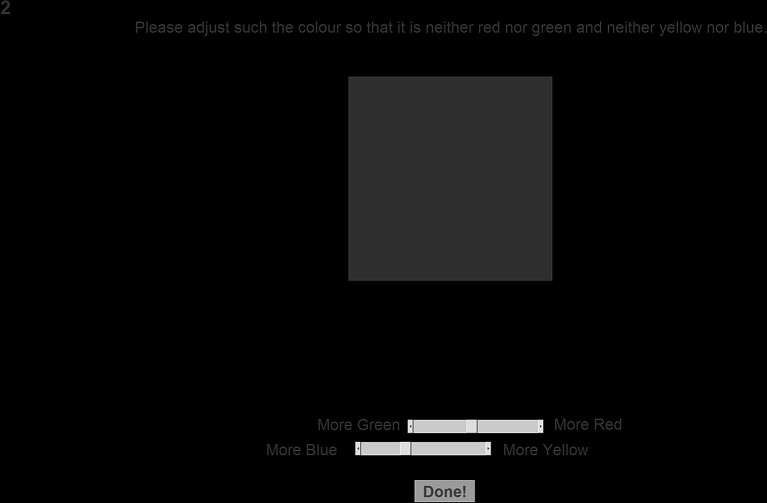
Screenshot of the neutral gray task. This figure has been cropped to improve readability.

The neutral gray task involved asking the observers, “Please adjust the color patch so that it is neither red nor green and neither yellow nor blue” (Chauhan et al., 2014). Five repetitions were taken for each condition resulting in 25 recorded neutral gray adjustments for each observer. The observer’s final neutral gray selections were saved in RGB format, then corrected and converted back into the CIELAB color space before any analyses took place. This color space was chosen due to its approximate perceptual uniformity; see (Luo, 2016) for more information. The colors were corrected to better reflect the color that was presented on the screen during the experiment, as the actual and requested colors may differ depending on the monitor’s screen settings (e.g., brightness). When the observers were adjusting the color patch, they had two sliders available to them, one that changes the green to red (a*) value and another that changes the blue to yellow (b*) value. The RGB values of the observer’s final neutral gray selections were saved. Before any analyses took place, the monitor’s native RGB color space was analyzed using a spectroradiometer (SpectraScan^®^ Spectroradiometer PR-670). The RGB values of the neutral gray selections of the participants were then converted into the CIELAB color space using a transformation from the monitor’s calibration. This was done so that any color values reported in this section better reflect the colors displayed on the computer screen when the observers were making their judgments. See Figure 2 for a screenshot of the neutral gray task. See Section “Color stimuli” for information on the color stimuli.

### 2.4. Classification task

After the neutral gray selection task was completed for each condition, the observer was asked to identify the odor that was presented. This task involved selecting the odor the observer thought was presented from a pre-compiled list of 26 different options; one option was “Can not identify,” and the remaining 25 elements were different odors, presented in alphabetical order. These odors are basil, camphor, caramel, cherry, chocolate, cinnamon, coffee, eucalyptus, freshly cut grass, ginger, grapefruit, lavender, lemon, lime, onion, orange, pepper, peppermint, pine, rose, sage, vanilla, wood, and ylang ylang.

### 2.5. Olfactory stimuli

**Figure 3:**
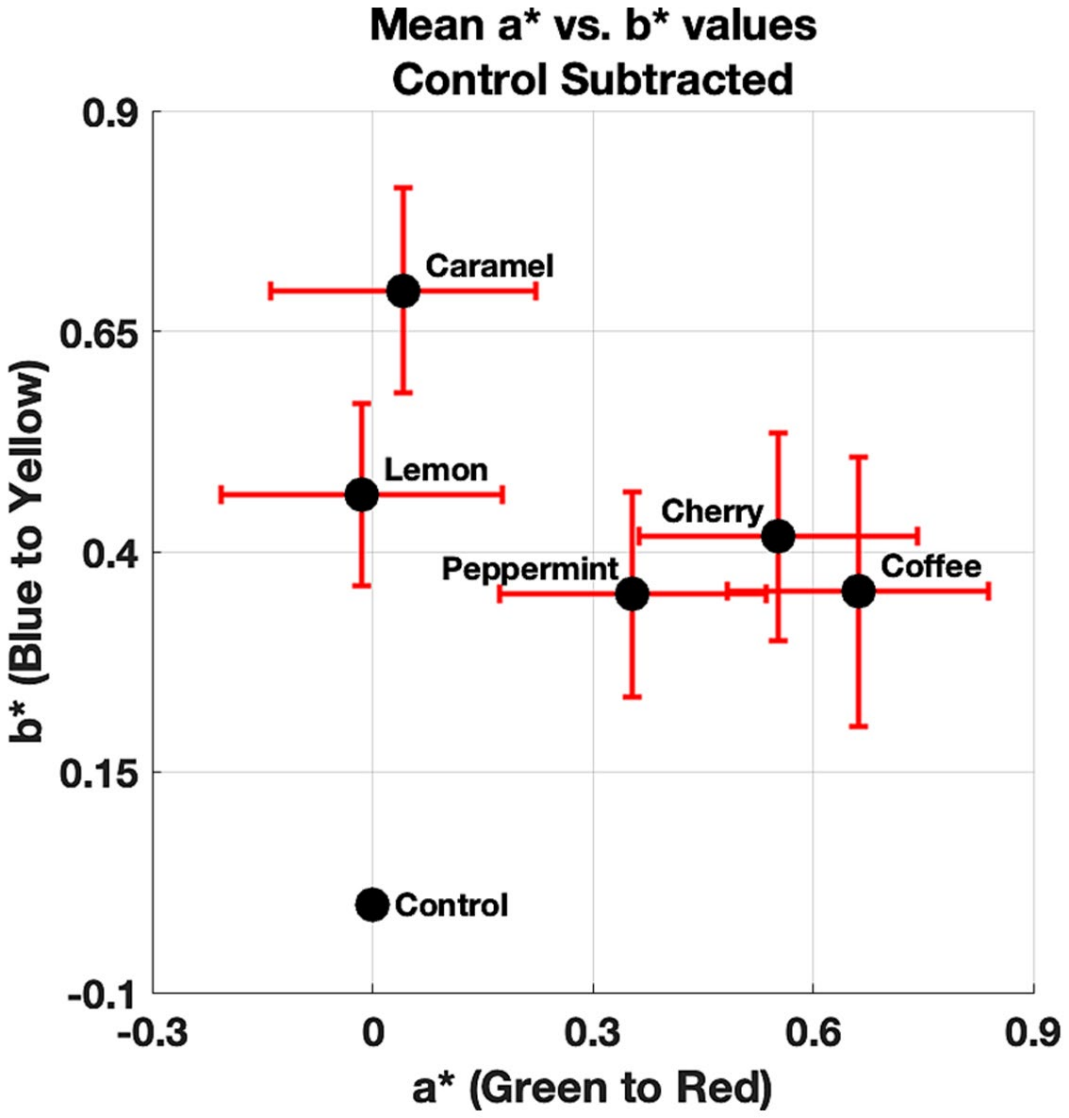
Mean a* and b* values for each of the odors and the control. Error bars denote standard mean error for the a* (*x*-axis) and b* (*y*-axis) dimensions.

Five different odors were used in the experiment; two from Miaroma™ (lemon and peppermint) and three from Mystic Moments™ (coffee, caramel, and cherry). These odors were chosen as they induced the most robust odor-color correspondences in our prior work, see Ward R. J. et al., (2021; Figure 3B). In addition to these odors, plain, unscented water was used as a control. The odors were presented to the users using ultrasonic diffusers. The diffusers were marked with a random number between one and six; this aligned with the number presented on a computer screen during the experiment. This number was for the experimenter to indicate what odor to present to the observer. The experimenter operated the diffusers which were hidden from the observer behind a divider ≈ 1 meter away to the observer’s left. To create the olfactory stimuli five drops of the respective essential oil were added to 500 mL of tap water approximately 20 min before the experiment started. To avoid olfactory contamination each diffuser was only used for a singular odor, and the hole in the diffuser was taped over to minimize any odor leakage.

### 2.6. Color stimuli

The monitor used for the experiments was a calibrated 28” BenQ GL2450-B. The lightness of the presented stimuli inside of MATLAB was fixed at L* = 50 and could not be adjusted by the observer. The color patch was presented in the center of the screen horizontally and consisted of a 500 × 500 pixel patch and was presented at a resolution of 1080p. All text on the screen was adjusted to be the same color as the color patch; this included the instructions, the number corresponding to an odor, the slider labels, and the “Done” button. The application’s background color was black; (*X* = 0.1496, *y* = 0.1360, *z* = 0.2763) after correction. The sliders were visually offset on the *x*-plane to stop the participants from simply centering the sliders together rather than aligning them based on the color patch. The starting color was set to be randomly between −5 and + 5 for the a* and b* channels separately. The starting position of the sliders was also adjusted to compensate. The range of −5 and + 5 was chosen to give the observers finer control over their final neutral gray selections, as a larger range would have resulted in larger jumps between colors when adjusting the slides. Larger jumps in the slides would make a truly achromatic stimuli harder to obtain as they might miss the values required for the task. As all external illumination was blacked out D65 was used as the white point as the observers were already dark-adapted. D65 is a CIE standard device-independent illuminate that simulates noon daylight and is commonly used in the relevant literature (Maric and Jacquot, 2013; Chauhan et al., 2014; Nehmé et al., 2016; Cuskley et al., 2019) as it makes it easy for the results to be reproducible.

### 2.7. Data analysis

The data analysis, figure preparation, and the programming of the experiment were performed in MATLAB 2021b. The CircStat toolbox (Berens, 2009a) was used for the circular statistics.

## 3. Results

First, the mean value for each of the observer’s a* and b* color ratings across repetitions was calculated to reduce the signal-to-noise ratio of the reported neutral gray selections. Hue angles were then calculated using the a* and b* values collapsed across repetitions. Rayleigh tests were conducted (Bonferroni–Holm corrected) on these hue angles for each condition (all of the odors and the control). Circular data involves measurements that wrap around itself circularly and therefore, requires appropriate statistical tests that consider the circular nature of underlying data. The Rayleigh test (Fisher, 1995; Berens, 2009b) checks for the non-uniformity of circular data. In other words, it tests to see if the data points are evenly distributed around a circle. The null hypothesis is that the population is uniformly distributed around a circle, indicating a random distribution (chance selection). The alternate hypothesis is that the population is not uniformly distributed around a circle, indicating a non-random distribution (not attributed to chance). The Rayleigh test is often denoted as *Z* or *R*, which refers to the vector sum of the numbers representing the data points on the unit circle. Rayleigh tests indicated that all of our conditions induced a non-random distribution; caramel (*Z* = 22.37, *p* < 0. 05), cherry (*Z* = 22.05, *p* < 0.05), coffee (*Z* = 22.20, *p* < 0.05), peppermint (*Z* = 22.26, *p* < 0.05), lemon (*Z* = 21.68, *p* < 0.05), and the control (*Z* = 22.78, *p* < 0.05). In other words, the neutral gray selections for all of our conditions differed from chance selection. Next, we proceeded to correct for the control by subtracting the raw a* and b* values for each of the odors from the observer’s rating for the control. The mean a* and b* values after correcting for the control for each condition is shown in Figure 3.

**Figure 4:**
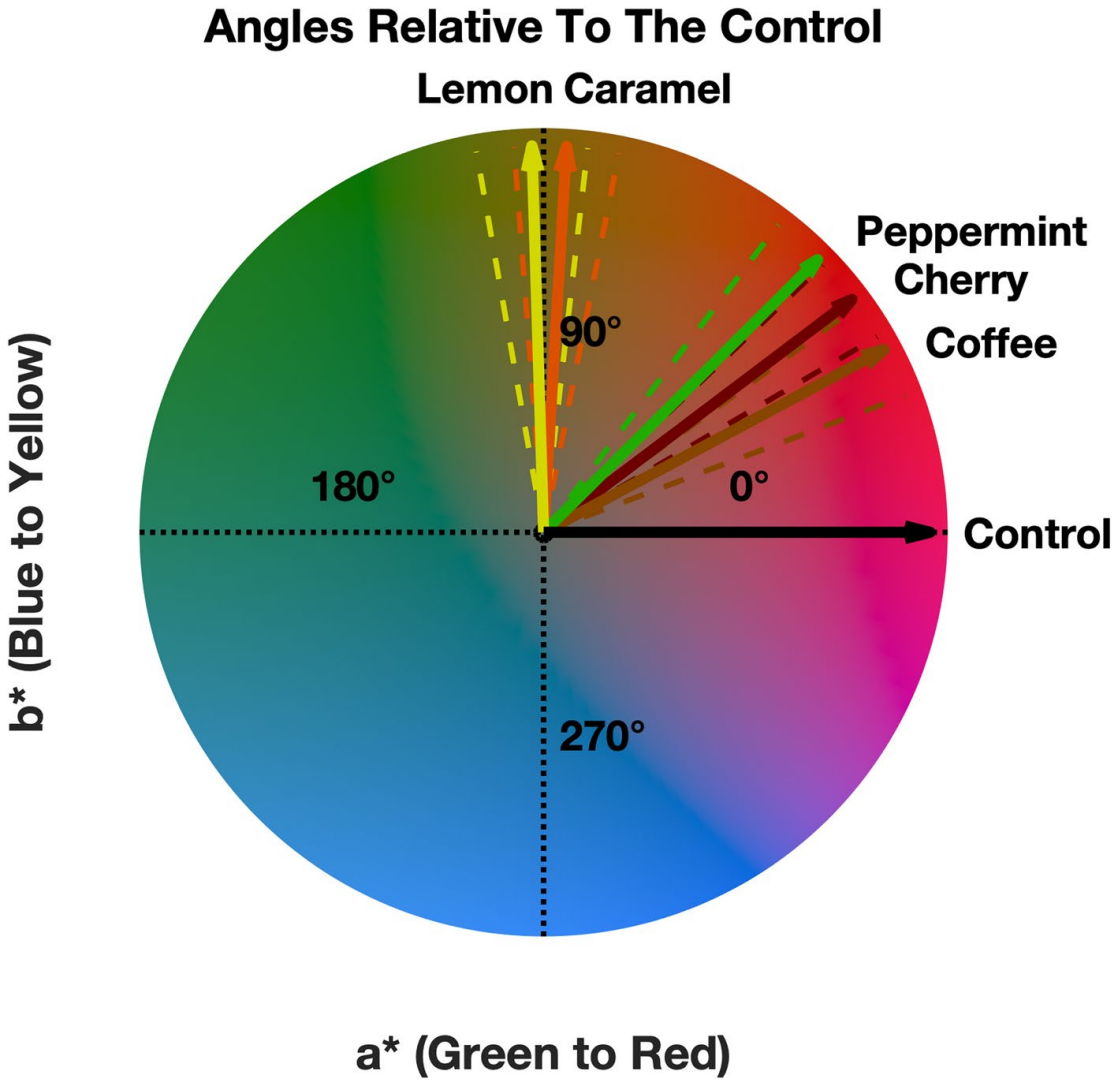
Angle for each of the odors relative to the control. Error bars (dashed lines) denote the standard mean error.

From Figure 3, a pattern is observed that suggests a small effect on the presence of odors influencing the observer’s neutral gray selections. Figure 3 also shows that this shift in the CIELAB color space is generally small (less than one unit). Next, we decided to compare the direction of the mean a* and b* values with respect to the control in the CIELAB color space. This was done to visually see if there was a systematic effect. That is, does the presence of the odors shift the perceived neutral gray point toward their anticipated color correspondences? The angles relative to the control for the mean a* and b* are shown in Figure 4.

Figure 4 suggests that the angles all point toward warm colors, indicating that the presence of any of our odors induced a general shift to warmer colors, which is independent of the presented odor. Figure 4 shows that four (lemon, caramel, cherry, and coffee) out of five odors roughly trend toward their anticipated crossmodal correspondences except for peppermint, which we initially expected to be trending toward a green-blue color. See the discussion for more information about Figure 4. Next, we proceeded to statistically validate these findings.

We first tested to see if these results could be attributed to a spatial response bias due to the x-axis offset of the slider’s visual location on the screen. A two-sample t-test was conducted on the control corrected a* and b* values collapsed across odors. This revealed no significant difference between the a* and b* values *t*(119) = 0.5296, *p* = 0.69, suggesting that if a spatial response bias was present, it has been rectified when correcting for the control. Now, it is known that there is no bias in the underlying data; hue angles were then calculated using the a* and b* values that have been corrected for the control (control shifted angle matrix). To determine if the hue angles of all of our odors were not attributed to chance after correcting for the control, Rayleigh tests (Bonferroni–Holm corrected) were then conducted on the control shifted angle matrix, revealing that the odors cherry (*Z* = 5.15, *p* < 0.05), coffee (*Z* = 5.23, *p* < 0.05), and peppermint (*Z* = 5.14, *p* < 0.05) induced a non-random distribution. The odors caramel (*Z* = 1.18, *p* > 0.05) and lemon (*Z* = 0.36, *p* > 0.05) were randomly distributed. In other words, the neutral gray selections for cherry, coffee, and peppermint demonstrated a systematic effect and did not originate from chance selection; the inverse could be said about caramel and lemon. However, as the odors may have multiple color correspondences, for instance, lemon is consistently associated with various shades of yellow, green, and pink (Ward R. J. et al., 2021), we decided to take this finding lightly. Pair-wise *post-hoc V*-tests were conducted to determine if the odors shared the same mean direction as the other odors and the control. The *V*-test is similar to the Rayleigh test with a difference in the alternative hypothesis, which is assumed to have a known mean direction (Zar, 1999; Berens, 2009b). The null hypothesis is that the data points are uniformly distributed around a circle. The alternate hypothesis is that the data points are not uniformly distributed around a circle and have a pre-specified mean angle. The *V*-test has more power than the Rayleigh test if there is a reason to believe there is a specific mean direction. The *V* test is often denoted as *V*, which refers to the multiplication of the Rayleigh test with the cosine of the difference between the actual and anticipated mean direction. *Post-hoc* pair-wise *V*-tests (Bonferroni–Holm corrected) were conducted between all odors and the control. Most importantly, this revealed that caramel (*V* = 7.23, *p* > 0.05), cherry (*V* = 19.55, *p* > 0.05), peppermint (*V* = 20.10, *p* > 0.05), and lemon (*V* = 6.52, *p* > 0.05) do not share the same mean direction as the control. However, the mean direction for coffee (*V* = 23.26, *p* < 0.05) did overlap with the control. Suggesting that the presence of some odors, but potentially not all, may modulate color appearance. See Supplementary Table S1 for all of the results for the pair-wise comparisons.

To further validate this finding, pair-wise *t*-tests (Bonferroni–Holm corrected) were conducted on distance matrices. These distance matrices are the Euclidean distance from each condition to every other condition. The results indicate that all odors are significantly different from the control, and all conditions are significantly different from each other (all *p*-values less than 0.05). Zero was used as the hypothesized mean, as zero indicates both odors occupy the same space. Therefore, providing further evidence that the presence of odors could modulate color appearance. See Supplementary Table S2 for all of the results for this pair-wise comparison.

In terms of correct identification of the presented odors, caramel was correctly identified 33% of the time, with cherry being 33%, coffee at 12%, peppermint at 33%, and lemon at 12%, indicating the observers had trouble correctly labeling the odors. However, odor naming, even for common odors, has been shown to be a difficult task but is typically accompanied by a strong feeling of knowing (Jönsson et al., 2005).

In summary, these results suggest that the presence of different odors influenced the observer’s neutral gray selections and that the angles of the odors trend toward warmer colors. Based on Figure 4, four (lemon, caramel, cherry, and coffee) out of five of the odors shift the perceived neutral gray point toward the color that is associated with the respective odor (Ward R. J. et al., 2021) except for peppermint.

## 4. Discussion

Our hypothesis was that the presence of odors would influence the perception of color, with observers overcorrecting their neutral gray selections to counteract their odor-color correspondences. The second half of this hypothesis did not hold; however, we did find that the presence of any of our odors induced a shift toward warmer colors, supporting the first half of our hypothesis. For the second half of our hypothesis, four (lemon, caramel, coffee, and caramel) out of five odors shifted the neutral gray point toward the anticipated crossmodal correspondences for the respective odor, indicating that our expectation that the observers would overcorrect to counteract this effect proved false. These findings demonstrate the effect that odors have on our color perception, specifically inducing a shift in a neutral gray setting.

Based on the crossmodal odor-color profiles from Ward R. J. et al. (2021; Figure 3B), caramel was typically associated with dark brown-yellow, coffee with dark brown and red, cherry with pink, red, and purple, peppermint with green and blue, and lemon with yellow, green, and pink. Here we found that caramel induced a shift in the neutral gray point toward a yellow-brown, cherry and coffee toward a red-brown, peppermint toward a brown-red, and lemon toward yellow–green. With the exception of peppermint, we have shown that the presence of odors induced a shift roughly toward the expected crossmodal correspondences for each of the odors. The most popular explanation for the existence of odor-color correspondences is in terms of semantics (Spence, 2020b). It may be the case that our findings stem partly from a semantic basis, but at a perceptual level. That is, as crossmodal odor-color correspondences are strong and stable (Demattè et al., 2009) semantic knowledge of odor could have changed the observer’s perception of what a neutral gray is and due to the nature of the task presented to the observers, this lead us to conclude that this happened at a perceptual level, as they were asked to make a color patch achromatic, and there was no reference or language referring to the odors during the briefing or task. Therefore, suggesting that this effect does not occur at a decisional level.

In terms of cognitive penetrability, the idea that our expectations, thoughts, and beliefs can impact our perception of the world around us (Deroy, 2013) could play a role in our findings. That is, there might be top-down influences of higher cognitive processes that modify our perception, see (Delk and Fillenbaum, 1965; Macpherson, 2012; Deroy, 2013). Earlier research by Hansen et al. (2006) suggests that visual memory can modulate color appearance. Therefore, it could be the case that knowledge of the source of the odor penetrates/alters the perception of color. It is important to note that this knowledge of the identity of the odor could be induced by either a strong feeling of knowing (Jönsson et al., 2005) or by directly being able to name the odor. In turn, suggesting that odor-color correspondences are strong enough to influence color appearance. Stevenson et al. (2012) noted that when an odor is neither familiar nor nameable, odor-color correspondences may be modulated by intensity, hedonics, and/or irritancy. For odor-color correspondences, semantic attributes (identifiability and familiarity) are associated with more saturated colors and perceptual attributes (irritancy, intense, and unpleasant) are associated with brighter colors (Stevenson et al., 2012). It would stand to reason that, at least for some of our participants and potentially some of our odors, the semantic account might fail. In turn, suggesting that both semantic and perceptual attributes of the odors could also have played a role in explaining our findings. Crossmodal correspondences, in some cases, have been shown to be effected by emotions (Spence, 2020a; Di Stefano et al., 2021). Both unfamiliar odors (Schiffman, 1974; Benderly, 1988) and colors (Palmer et al., 2013; Gilbert et al., 2016) have been shown to be linked to emotions. Therefore, in the case of lack of knowledge of the identity of the odor the neutral gray selections could have been mediated by emotions.

Another, albeit more controversial, way of thinking about these findings, is in terms of crossmodal harmony. Crossmodal harmony refers to the way our senses complement and collaborate with other sensory modalities, such as audition (Spence and Di Stefano, 2022) and color perception (Judd and Wyszenk, 1975). In terms of olfaction, crossmodal harmony occurs when our sense of smell interacts and harmonizes with the other senses. Crossmodal harmony implies a balanced relationship between the component parts (Spence and Di Stefano, 2022). The experimental task could be conceived as a matching task between visual stimuli and ambient odors. It may be the case the perceived neutral gray selections differed due to crossmodal harmony, where the perceived neutral gray point shifted due to a harmony between the ambient odor and the visual stimuli. That is, the neutral gray selections have been modulated or matched in terms of pleasantness, or combinations of stimuli that were in a state of perceptual harmony.

A few limitations of this study should be mentioned. Firstly, the observers were restricted in adjusting the L* channel when asked to make an achromatic stimulus; this was done to make the experiment as simple as possible for the observer. However, including this additional dimension may supply more information to uncover why this effect occurs. For instance, it has been shown that when an odor cannot be named or is unfamiliar, the observer’s color matches may be modulated by the irritancy, intensity, and/or hedonics instead (Stevenson et al., 2012; Spence, 2020b). Considering that odor intensity and color lightness are correlated sensory dimensions (Kemp and Gilbert, 1997), the inclusion of this additional dimension may help uncover if there is any involvement from the intensity of the presented odor. Another limitation of the current study is the number of tested odors. We only tested five odors, and although we found that four out of five of the odors had a mean direction significantly different from the control, it would be beneficial to uncover the extent in which this bias is present and to uncover if the trend toward the expected crossmodal correspondences for a given odor is only true if it also happens to be associated with a warm color.

Future work could include investigating to see how the familiarity of the presented odors affects the observer’s ratings during the neutral gray task. That is, were the observers more biased if the presented odor was nameable or familiar compared to the observers where the presented odor was neither nameable nor familiar. To investigate this a larger sample size of observers would be required.

## 5. Conclusion

The results in this study suggest that the presence of an odor can bias an observer’s decision of what their perceived version of a neutral gray is. That is the hue angles relative to the control stimuli for all odors point toward warm colors. It was also found that the hue angles for four out of five of our odors had a mean direction significantly different from the control and all of the odors are not located in the same place in the CIELAB color gamut as each other. Indicating that the presence of some odors’ induces a shift of the perceived neutral gray point toward warmer colors and that four out of five of the odors are shifted toward their respective odor-color correspondences, further suggesting a small but systematic effect of the presence of odors on human color perception.

## Data availability statement

The raw data supporting the conclusions of this article will be made available by the authors, without undue reservation.

## Ethics statement

The studies involving humans were approved by the Department of Psychology, University of Liverpool. The studies were conducted in accordance with the local legislation and institutional requirements. The participants provided their written informed consent to participate in this study.

## Author contributions

RW conceived and designed the experiments, performed the experiments, analyzed and interpreted the data, and wrote the paper. MA performed the experiments and wrote the paper. SW conceived and designed the experiments, interpreted the data, wrote the paper, provided supervisory support, and provided materials and reagents. AM wrote the paper and provided supervisory support. All authors contributed to the article and approved the submitted version.

## Conflict of interest

The authors declare that the research was conducted in the absence of any commercial or financial relationships that could be construed as a potential conflict of interest.

## Publisher’s note

All claims expressed in this article are solely those of the authors and do not necessarily represent those of their affiliated organizations, or those of the publisher, the editors and the reviewers. Any product that may be evaluated in this article, or claim that may be made by its manufacturer, is not guaranteed or endorsed by the publisher.
