# Supplementary material for: Odors modulate color appearance
Source: Front Psychol. 2023 Oct 6;14:1175703. doi: 10.3389/fpsyg.2023.1175703 (PMC10587423; doi:10.3389/fpsyg.2023.1175703)
Supplement: Supplementary file 1 [file Table_1.DOCX]

| **V Test Between** | **Statistics (V stat, corrected p value)** | **Conclusion** |
| --- | --- | --- |
| Caramel – Control | 7.23, p > 0.05 | The odour caramel does not share the same mean direction as the mean direction for the control. |
| Cherry - Control | 19.55, p > 0.05 | The odour cherry does not share the same mean direction as the mean direction for the control. |
| Coffee – Control | 23.26, p < 0.05 | The odour coffee shares the same mean direction as the mean direction for the control. |
| Peppermint – Control | 20.10, p > 0.05 | The odour peppermint does not share the same mean direction as the mean direction for the control. |
| Lemon – Control | 6.52, p > 0.05 | The odour lemon does not share the same mean direction as the mean direction for the control. |
| Cherry - Caramel | 24.08, p < 0.05 | Cherry shares the same mean direction as caramel. |
| Coffee – Caramel | 21.50, p < 0.05 | Coffee shares the same mean direction as caramel |
| Peppermint – Caramel | 23.79, p < 0.05 | Peppermint shares the same mean direction as caramel. |
| Lemon – Caramel | 3.15, p > 0.05 | Lemon does not share the same mean direction as caramel. |
| Control - Caramel | 72.85, p < 0.05 | The control shares the same mean direction as caramel. |
| Caramel – Cherry | 11.53, p < 0.05 | The odour caramel shares the same mean direction as cherry. |
| Coffee – Cherry | 24.02,p < 0.05 | Coffee shares the same mean direction as cherry. |
| Peppermint – Cherry | 24.82, p < 0.05 | Peppermint shares the same mean direction as cherry. |
| Lemon – Cherry | 4.5, p < 0.05 | The odour lemon shares the same mean direction as cherry. |
| Control – Cherry | 94.31, p < 0.05 | The control shares the same mean direction as cherry. |
| Caramel – Coffee | 10.22, p > 0.05 | Caramel does not share the same mean direction as coffee. |
| Cherry - Coffee | 23.85, p < 0.05 | Cherry shares the same mean direction as coffee. |
| Peppermint – Coffee | 24.07, p < 0.05 | Peppermint shares the same mean direction as coffee. |
| Lemon – Coffee | 5.68, p < 0.05 | Lemon shares the same mean direction as coffee. |
| Control – Coffee | 111.45, p > 0.05 | The control does not share the same mean as coffee. |
| Caramel – Peppermint | 11.40, p > 0.05 | Caramel does not share the same mean direction as peppermint. |
| Cherry - Peppermint | 24.85, p < 0.05 | Cherry shares the same mean direction as peppermint. |
| Coffee – Peppermint | 24.28, p < 0.05 | Coffee shares the same mean direction as peppermint. |
| Lemon – Peppermint | 4.68, p > 0.05 | Lemon does not share the same mean direction as peppermint. |
| Control – Peppermint | 97.14, p < 0.05 | The control shares the same mean direction as peppermint. |
| Caramel – Lemon | 5.68, p > 0.05 | Caramel does not share the same mean direction as lemon. |
| Cherry - Lemon | 16.94, p > 0.05 | Cherry does not share the same mean direction as lemon. |
| Coffee – Lemon | 21.55, p < 0.05 | Coffee shares the same mean direction as lemon. |
| Peppermint – Lemon | 17.62, p > 0.05 | Peppermint does not share the same mean direction as lemon. |
| Control – Lemon | 118.57, p < 0.05 | The control shares the same mean direction as lemon. |

Table S1. Pair wise V test results conducted to determine which odours do not share the same mean direction.

| **t-tests between** | **Statistics (t-value, p-value)** | **Conclusion** |
| --- | --- | --- |
| Control - Caramel | t = 6.99, p < 0.05 | All odours and the control do not occupy the same space as any another odour. |
| Control - Cherry | t = 7.18, p < 0.05 |  |
| Control - Coffee | t = 7.82, p < 0.05 |  |
| Control - Peppermint | t = 7.22, p < 0.05 |  |
| Control - Lemon | t = 6.43, p < 0.05 |  |
| Cherry - Caramel | t = 7.09, p < 0.05 |  |
| Coffee – Caramel | t = 9.09, p < 0.05 |  |
| Peppermint – Caramel | t = 8.96, p < 0.05 |  |
| Lemon – Caramel | t = 9.97, p < 0.05 |  |
| Control - Caramel | t = 6.99, p < 0.05 |  |
| Caramel – Cherry | t = 7.09, p < 0.05 |  |
| Coffee – Cherry | t = 5.96, p < 0.05 |  |
| Peppermint – Cherry | t = 6.87, p < 0.05 |  |
| Lemon – Cherry | t = 7.49 p < 0.05 |  |
| Control – Cherry | t = 7.18, p < 0.05 |  |
| Caramel – Coffee | t = 9.09, p < 0.05 |  |
| Cherry - Coffee | t = 5.96, p < 0.05 |  |
| Peppermint – Coffee | t = 7.21, p < 0.05 |  |
| Lemon – Coffee | t = 7.51, p < 0.05 |  |
| Control – Coffee | t = 7.82 p < 0.05 |  |
| Caramel – Peppermint | t = 8.96, p < 0.05 |  |
| Cherry - Peppermint | t = 6.87, p < 0.05 |  |
| Coffee – Peppermint | t = 7.21, p < 0.05 |  |
| Lemon – Peppermint | t = 6.90, p < 0.05 |  |
| Control – Peppermint | t = 7.22, p < 0.05 |  |
| Caramel – Lemon | t = 9.97, p < 0.05 |  |
| Cherry - Lemon | t = 7.49, p < 0.05 |  |
| Coffee – Lemon | t = 7.51, p < 0.05 |  |
| Peppermint – Lemon | t = 6.90, p < 0.05 |  |
| Control – Lemon | t = 6.43, p < 0.05 |  |

Table S2. Pairwise t-test results to see if the distance from one odour to another are the same. The degrees of freedom for all tests in this table is 23.
